# Area-Specific Synapse Structure in Branched Posterior Nucleus Axons Reveals a New Level of Complexity in Thalamocortical Networks

**DOI:** 10.1523/JNEUROSCI.2886-19.2020

**Published:** 2020-03-25

**Authors:** Javier Rodriguez-Moreno, Cesar Porrero, Astrid Rollenhagen, Mario Rubio-Teves, Diana Casas-Torremocha, Lidia Alonso-Nanclares, Rachida Yakoubi, Andrea Santuy, Angel Merchan-Pérez, Javier DeFelipe, Joachim H.R. Lübke, Francisco Clasca

**Affiliations:** ^1^Department of Anatomy and Neuroscience, School of Medicine, Autónoma de Madrid University, 28029 Madrid, Spain,; ^2^Institute of Neuroscience and Medicine INM-10, Research Centre Jülich GmbH, 52425 Jülich, Germany,; ^3^Laboratorio Cajal de Circuitos Corticales, Centro de Tecnología Biomédica, Universidad Politécnica de Madrid, Pozuelo de Alarcón, 28223 Madrid, Spain,; ^4^Instituto Cajal, Consejo Superior de Investigaciones Científicas, Arce 37 28002, Madrid, Spain,; ^5^CIBERNED, Centro de Investigación Biomédica en Red de Enfermedades Neurodegenerativas, 28031 Madrid, Spain,; ^6^Departamento de Arquitectura y Tecnología de Sistemas Informáticos, Universidad Politécnica de Madrid. Boadilla del Monte, 28660 Madrid, Spain,; ^7^Department of Psychiatry, Psychotherapy and Psychosomatics, Medical Faculty RWTH University Hospital Aachen, 52074 Aachen, Germany, and; ^8^JARA-Translational Brain Medicine, 52425 Jülich-Aachen, Germany

**Keywords:** 3D electron microscopy, mitochondria, motor cortex, somatosensory cortex, synapse, thalamus

## Abstract

Thalamocortical posterior nucleus (Po) axons innervating the vibrissal somatosensory (S1) and motor (MC) cortices are key links in the brain neuronal network that allows rodents to explore the environment whisking with their motile snout vibrissae. Here, using fine-scale high-end 3D electron microscopy, we demonstrate in adult male C57BL/6 wild-type mice marked differences between MC versus S1 Po synapses in (1) bouton and active zone size, (2) neurotransmitter vesicle pool size, (3) distribution of mitochondria around synapses, and (4) proportion of synapses established on dendritic spines and dendritic shafts. These differences are as large, or even more pronounced, than those between Po and ventro-posterior thalamic nucleus synapses in S1. Moreover, using single-axon transfection labeling, we demonstrate that the above differences actually occur on the MC versus the S1 branches of individual Po cell axons that innervate both areas. Along with recently-discovered divergences in efficacy and plasticity, the synaptic structure differences reported here thus reveal a new subcellular level of complexity. This is a finding that upends current models of thalamocortical circuitry, and that might as well illuminate the functional logic of other branched projection axon systems.

**SIGNIFICANCE STATEMENT** Many long-distance brain connections depend on neurons whose branched axons target separate regions. Using 3D electron microscopy and single-cell transfection, we investigated the mouse Posterior thalamic nucleus (Po) cell axons that simultaneously innervate motor and sensory areas of the cerebral cortex involved in whisker movement control. We demonstrate significant differences in the size of the boutons made in each area by individual Po axons, as well as in functionally-relevant parameters in the composition of their synapses. In addition, we found similarly large differences between the synapses of Po versus ventral posteromedial thalamic nucleus axons in the whisker sensory cortex. Area-specific synapse structure in individual axons implies a new, unsuspected level of complexity in long-distance brain connections.

## Abstract (precis)

Thalamocortical posterior nucleus (Po) axons innervating the vibrissal somatosensory (S1) and motor (MC) cortices are key links in the brain neuronal network that allows rodents to explore the environment whisking with their motile snout vibrissae.

## Introduction

Rodents explore their environment by rhythmically “whisking” with their motile facial vibrissae. Time-dependent correlations between motor commands and vibrissal follicle mechanoreceptor signals are used for inferring object position and texture. Such computations are performed by multilevel closed-loop neuronal networks encompassing the brainstem, thalamus, and neocortex (for review, see Ahissar and Oram, 2015).

Two thalamocortical pathways lay at the core of these loops: the ventral posteromedial thalamic nucleus (VPM) axons that innervate focally the L4 the “barrel” domains of the vibrissal primary somatosensory cortex (S1), and the posterior thalamic nucleus (Po) axons that innervate mainly L5a but also L1 of S1 (Wimmer et al., 2010; Casas-Torremocha et al., 2019). Importantly, the Po axons may target, in addition, the motor cortex (MC) middle layers (L5a–L3; Ohno et al., 2012; Hooks et al., 2015; Casas-Torremocha et al., 2019). VPM axons relay time-locked mechanoreceptive single-whisker trigeminal signals, crucial for computing object location. In turn, Po axons convey information mainly about timing/amplitude differences between ongoing cortical outputs and multi-whisker sensory signals, which may be important for computing whisker position (Yu et al., 2006; Groh et al., 2014; Ahissar and Oram, 2015).

Activation of S1–L4 VPM synapses elicits large currents in cortical neurons (Lee and Sherman, 2008; Petreanu et al., 2009) and drives their firing with low failure rates (Gil et al., 1999; Bruno and Sakmann, 2006). VPM synapses are driven only by ionotropic receptors, depress rapidly upon repetitive stimulation (Lee and Sherman, 2008) and, after an early postnatal period, show considerable resistance to sensory experience-dependent changes (Audette et al., 2019). In contrast, synaptic potentials evoked in S1 by Po axons show slower rise and decay times, and elicit smaller currents (Bureau et al., 2006; Petreanu et al., 2009). The Po S1 synapses involve both ionotropic and metabotropic glutamate conductances, show paired-pulse facilitation (Petreanu et al., 2009; Viaene et al., 2011) and rapidly increase their efficacy in response to learned reward even in the adult (Audette et al., 2019). Recent data about the Po MC synapses indicate that, surprisingly, they elicit large currents, depress upon repetitive activation (Hooks et al., 2015; Mo and Sherman, 2019) and involve only ionotropic receptors (Casas-Torremocha et al., 2019).

In cortical synapses, bouton and mitochondrial volume, synaptic vesicle pool size, as well as postsynaptic density (PSD) complexity and surface area directly correlate with increased neurotransmitter release probability and synaptic strength (Geinisman, 1993; Matz et al., 2010; Holderith et al., 2012). Furthermore, the PSD surface area is proportional to the number of postsynaptic receptors (Nusser et al., 1998; Kharazia and Weinberg, 1999; Ganeshina et al., 2004; Tarusawa et al., 2009). Thus, given the markedly divergent effect observed in S1 versus MC Po synapses, and recent light microscope evidence that Po S1 axon varicosities are smaller than those in MC (Casas-Torremocha et al., 2019) we aimed to determine whether Po synapses in MC and S1 varied in their ultrastructural composition. Moreover, as studies in rat have reported that MC and S1 can be simultaneously targeted by branches of the same individual Po axons (Noseda et al., 2011; Ohno et al., 2012), we also set out to elucidate if structural differences occur between boutons on separate branches of the same Po cell axon.

To measure identified Po synapses in S1 and MC, we combined single-cell and bulk axonal labeling with light microscopy and fine-scale 3D-electron microscopy [serial sectioning transmission electron microscopy (ssTEM), and focused ion beam milling scanning electron microscopy (FIB-SEM)]. In addition, as Po and VPM synapses have been recently shown to elicit marked different effects on S1 neurons, we reanalyzed our own dataset on VPM S1 synapses (Rodriguez-Moreno et al., 2018) and compared it with the Po synapse data. Axonal varicosities of Po individual axons in MC are significantly larger than those of the same axons in S1, and the composition of their synapses, strikingly different. Similar differences exist between Po and VPM axon synapses in different layers of S1.

## Materials and Methods

### 

#### 

##### Animals and anesthetic procedures.

Experiments were performed on adult (60–105 d old; 25–32 g body weight) male C57BL/6 mice bred in the animal facilities of the School of Medicine of the Autónoma de Madrid University. All procedures involving live animals were conducted under protocols approved by the University ethics committee and the competent Spanish Government agency (PROEX175/16), in accordance with the European Community Council Directive 2010/63/UE. Animals were housed in pairs in cages containing some toys, and provided chow and water *ad libitum* under a 12 h light/dark cycle. Efforts were made to minimize the number of animals required. Six male mice (60–65 d old) were used for the experiments aimed at biotinylated dextranamine (BDA) population-labeling of Po axons for light and electron microscopy. Thirty-two further mice were electroporated with Sindbis Pal-eGFP RNA to individually label thalamic neurons and reveal the full extent of their cell axons.

For both types of experiment, mice were first anesthetized with an initial intraperitoneal injection of ketamine (0.075 mg/g body weight) and xylazine (0.02 mg/g body weight), and then maintained with oxygenated isoflurane (0.5–2%) throughout the surgical procedure. At the end of the surgery, the isoflurane flow was interrupted and animals recovered promptly. Ibuprofen (120 mg/L) was added to the drinking water to ensure analgesia during the survival period. At the time of kill, animals were overdosed with sodium pentobarbital (0.09 mg/g body weight, i.p.).

##### BDA iontophoresis for selective population-labeling of Po axons.

Animals were placed in a stereotaxic frame (Kopf Instruments). BDA (10,000 Da; 2.5% in saline; Invitrogen) was iontophoretically microinjected (positive tip, 0.7–0.8 μA; 1 s on/off cycle, 30–40 min duration) with a Midgard Precision Current Source (Stoelting) under stereotaxic guidance (1.8 mm posterior, 1.3 mm lateral, and 2.7 mm ventral to bregma; Paxinos and Franklin, 2012), using borosilicate micropipettes (WPI; outer tip diameter: 7–10 μm). The micropipette was finally removed, and the muscle and skin were sutured.

##### Tissue processing of the BDA-injected brains.

Following a 5 d survival, animals were perfused with PBS (0.1 m) followed by 4% paraformaldehyde (PFA) + 0.1% glutaraldehyde in 0.1 m PB for 30 min. Brains were removed from the skull, and postfixed by immersion for 1 h at 4°C in the same solution. Two series of parallel coronal sections (50 μm thickness) were cut with a Leica VT 1200S vibratome (Leica Microsystems). Sections were cryoprotected by incubation in a sucrose solution (30% in 0.1 m PB) overnight, and were then rapidly freeze-thawed in liquid nitrogen (1 min) and stored in PB until further use.

**Figure 1.**
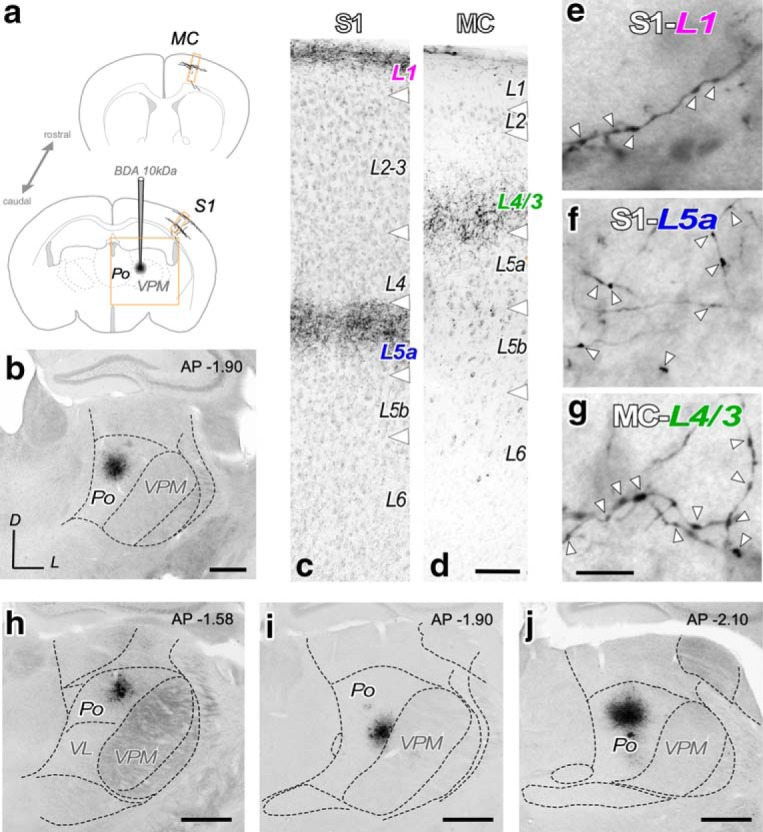
Selective bulk-labeling of Po thalamocortical axons in the vibrissal MC and S1. ***a***, BDA iontophoresis procedure. Schematic coronal brain sections depicting the BDA deposit in Po, and the anterogradely labeled thalamocortical axonal projection in S1 and MC. ***b***, A typical iontophoretic BDA Po deposit. The region illustrated is the area framed in ***a***. Thionin counterstain. Scale bar, 250 μm. ***c***, ***d***, Coronal sections showing the layer selective arborization of BDA-labeled Po axons in specific layers, namely L1 and L5a in S1 (***c***) and L4/3 in MC (***d***). Scale bar, 100 μm. ***e***–***g***, High-magnification images of BDA-labeled Po axons in S1–L1 (***e***), in S1–L5a (***f***), and in MC-L4/3 (***g***). Axonal varicosities are marked by arrowheads. Scale bar, 10 μm. ***h***–***j***, Coronal sections displaying the center of three BDA deposit in Po used in this study. Distance to bregma (in mm) is indicated t the top right. Nuclear boundaries were delineated over the cytochrome-oxidase (***h***) or thionin (***i***, ***j***) counterstains. Scale bar, 250 μm. VL: Ventral Lateral Thalamic Nucleus.

In the first series of sections, peroxidase activity was blocked by incubation in PB-buffered H_2_O_2_ for 10 min, and sections were then incubated in an avidin-biotin-peroxidase kit (1:100; Vectastain Elite, Vector Laboratories) diluted in PB. After washing in PB, labeling was visualized using the glucose oxidase-3–3′diaminobenzidine (DAB) nickel sulfate-enhanced method (Shu et al., 1988). Sections were mounted onto gelatin-coated glass slides, counterstained with thionin, dehydrated in graded ethanol, defatted in xylene, and coverslipped with DePeX (Serva). These sections were used for light microscopic analysis (Nikon Eclipse 600; 4–40× objectives) of the cytoarchitectonic localization of the injection site in the thalamus and the anterogradely labeled axons in the neocortex. Of the 12 BDA injected hemispheres, four showed BDA injections restricted to Po and robust axonal labeling in S1 and MC were selected (Fig. 1). The cortical areas and layers were identified based on the thionin counterstaining of the sections. The vibrissal region of the MC as defined in this study is located along the border between cytoarchitectonic areas AgM/M2 and AgL/M1 of the frontal cortex (bregma AP: 0.5–2.2 mm, ML: 1–1.5 mm; Hooks et al., 2015; Casas-Torremocha et al., 2019). The vibrissal domain of S1 is readily identifiable on the thionin-stained sections by its L4 barrel cell clusters.

The second series of sections followed a protocol identical to that described above, except for the omission of the oxygen-peroxide blocking step and the absence of the nickel sulfate-enhancement in the glucose oxidase-DAB reaction. In the four injection experiments that produced optimal labeling of Po axons (Figure 1) free-floating sections from this series containing the regions with the cortical labeling underwent further processing for electron microscopy. In these sections, the glucose oxidase-DAB reaction was followed by incubation in 1% osmium tetroxide (Electron Microscopy Science) diluted in PB for 45 min at room temperature. Following thorough washing in PB, sections were first rinsed in 50% ethanol, incubated for 40 min in 1% uranyl-acetate diluted in 70% ethanol in the dark, and dehydrated in an ascending series of ethanol to absolute ethanol. The dehydrated sections were transferred to acetonitrile (Scharlab), and then transferred to an epoxy resin (Durcupan, Electron Microscopy Science) overnight. Finally, sections were flat-embedded in Durcupan and polymerized at 60°C for 48 h.

**Figure 2.**
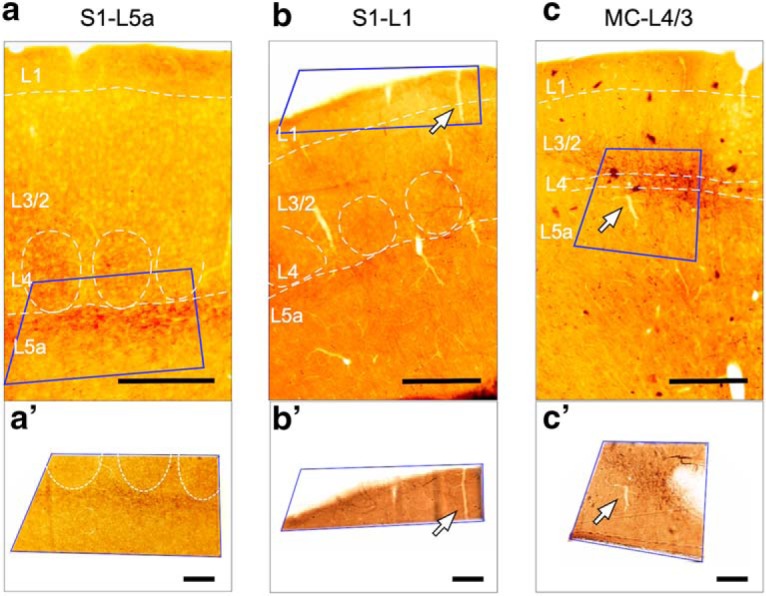
Selective sampling for EM of tissue areas containing BDA-labeled Po axon branches. ***a***–***c***, Resin-embedded coronal sections of the cortical areas to be examined with EM. The blue rectangle indicates the area that was cut out for subsequent EM serial sectioning. Scale bars: ***a′***–***c′***, 250 μm. An arrow identifies the same vascular landmark in both images. Scale bar, 100 μm.

After light microscopic inspection, samples containing dense labeling in the S1 and MC neocortex were cut out and glued onto pre-polymerized resin blocks for serial section (Fig. 2). Blocks prepared from the same experiments and tissue regions were used for subsequent ssTEM or FIB-SEM imaging (Table 1-1).

##### Tissue processing for ssTEM imaging of BDA-labeled Po boutons.

Embedded tissue blocks that contained L5a and L1 in S1 barrel cortex or L4/3 in MC were cut with a Leica Ultracut UCT ultramicrotome into serial 60 nm ultrathin sections (∼90 sections/series). They were collected on Pioloform-coated single-slot copper grids (Electron Microscopy Science). Thereafter, they were treated with lead citrate (3 min), and examined with a Libra 120 transmission electron microscope (EM; Carl Zeiss) equipped with a Proscan 2K bottom-mounted digital camera (Albert Tröndle) and the SIS Multi Image Acquisition software package (Olympus). At the EM level, BDA-labeled axons were easily identifiable by the opaque DAB reaction product. Serial digital images were taken at a magnification of 8000× and stored in a database until further use.

##### FIB-SEM 3D tissue preparation and imaging of BDA-labeled Po axons.

In addition, Durcupan-embedded tissue blocks from the same experiments were used to obtained 3D tissue samples using FIB-SEM. Here, a Crossbeam Neon40 EsB electron microscope equipped with a gallium FIB and a high-resolution scanning emission SEM column (Carl Zeiss) was used. To accurately select the regions-of-interest, a secondary electron microscopic image was acquired from the block surface that was overlaid and collated with previously obtained light microscopic images (Bosch et al., 2015). Once the appropriate location was chosen, a gallium ion beam was used to mill the sample to allow visualization of brain tissue under the block face on a nanometer scale. The recently milled surface was then imaged using a back-scattered electron detector (1.8 kV acceleration potential). The milling and imaging processes were sequentially repeated in an automated way, providing a stack of serial digital images that represented a 3D sample of the tissue (Merchán-Pérez et al., 2009). Image resolution in the *x–y* plane was 5 nm per pixel. Resolution in the *z*-axis (section thickness) was 20 nm, so the voxel size of the resulting image stack was 5 × 5 × 20 nm.

With those resolution parameters, images of 2048 × 1536 pixels (field-of-view of 10.24 × 7.68 μm) were obtained. A total of 20 different stacks of images of the neuropil in the three cortical regions-of-interest were obtained (7 stacks from S1-L5a, 6 from S1-L1, and 7 from MC-L4/3). The number of serial sections per stack ranged from 75 to 478; the total number of serial sections was 4874 (mean: 243.7 sections/stack). Registration (alignment) of serial sections was performed with the freely available Fiji software (Schindelin et al., 2012), using a rigid body model that allowed no deformation of individual images.

##### 3D-volume reconstruction and analysis of ssTEM and FIB-SEM image stacks.

The distribution in the cortex of thalamocortical axons is spatially selective in highly topographical patterns, in both the radial (laminar) and tangential dimensions of the cortex. Because we specifically wanted to examine the axons arising from Po labeled by means of small anterograde tracer deposits made in this nucleus, we focused on the cortical domains and layers that contained a maximum density of labeled thalamocortical axons (Fig. 2). Within these samples, the subsequent electron microscope analysis was essentially random: all labeled axon varicosities fully contained (ssTEM) or all labeled axon segments (FIB-SEM) that were visible within a given image stack were reconstructed and measured.

The 3D-volume reconstructions and measurements on ssTEM images were performed with the OpenCAR software (Contour Alignment Reconstruction; Sätzler et al., 2002). Digital images were aligned creating an image stack where all structures-of-interest were defined by closed contour lines of different color.

The *z*-stack image series acquired with FIB-SEM were 3D-segmented and measured with the Espina Interactive Neuron Analyzer software v2.1.10 (freely available at http://cajalbbp.es/espina/; Morales et al., 2011). 3D-reconstructions generated from separate *z*-stacks were digitally stitched using Unity 3D modeling software (Unity Technologies).

At each site the geometry of Po axon membrane, varicose or not, their mitochondria, and the postsynaptic membrane profile, its PSD and, when present, its spine apparatus or mitochondria were completely 3D reconstructed. From the membrane contours, 3D-volume reconstructions were performed, from which surface area and volume measurements were obtained. In many of the synaptic sites analyzed, the BDA-DAB staining complicated the visualization of individual vesicles. We thus counted the vesicles only in those varicosities (*n* = 29 in total) in which a lighter staining allowed unambiguous visualization of vesicles (a circular membrane contour around a paler core). We applied an unbiased stereological sampling method (physical dissector; Sterio, 1984; Sultan et al., 2002) for vesicle counting.

*En passant* synaptic boutons as the ones studied here are morphologically swellings in the axonal membrane which taper into the “inter-bouton” axon segments at both ends. For the measurements, we placed the “end” of the boutons at a transverse plane located where the main diameter became <0.5 μm. For consistency, all the bouton end delineations (in this and our previous VPM study; Rodriguez-Moreno et al., 2018) were performed by the same observer (J.R.-M.). Bouton volume and surface were measured in all the varicose axon segments, even those not containing a synaptic site. Varicosities that were visualized only partially because they traversed the image stack margins were not analyzed further.

In the sample of relatively long axon segments imaged using FIB-SEM we visualized, in addition to boutons, the complete inter-bouton axonal segments. These segments contained frequent (∼20–25%) synaptic sites that were located in smooth or just slightly swollen axon zones and contained some vesicles, yet lacked mitochondria. As placing ends would have been arbitrary, and the FIB-SEM sample of these synapses was small in number, the presynaptic element parameters (volume and surface) of these inter-bouton synapses were not measured.

##### Sindbis-pal-eGFP RNA electroporation for single-cell labeling.

To directly visualize the complete axonal tree of individual mouse Po neurons in an unambiguous manner, isolated Po neurons were transfected by means of *in vivo* electroporation (Porrero et al., 2016) of a RNA construct engineered to drive the expression of an enhanced green fluorescent protein (eGFP) fused with a palmytoilation motif from the growth-associated protein 43 (GAP43) under the Sindbis viral subgenomic promoter (Sind-Pal-eGFP; Furuta et al., 2001).

Micropipettes were pulled from Kwick-Fill borosilicate capillaries (1 mm outer diameter; WPI). Inner tip diameter was adjusted to 10–15 μm. To eliminate RNase activity, micropipettes were then kept in a stove overnight at 240°C and, after cooling, were backfilled with a RNA stock solution (1.8–2 μg/μl in 0.5 m NaCl) and mounted on a holder (WPI) that has both a pressure port and electrode connection. All procedures were performed over clean, single-use surfaces, and metal instrument tips were briefly exposed to a flame.

The micropipette tip was stereotaxically positioned into Po. Fifty to 100 nl of solution were injected using a precision electro-valve system (Picospritzer II, Parker Hannifin). Two to four 200 Hz trains of 1 ms negative-square pulses at 50 V were then applied through the micropipette tip using a CS20 stimulator (Cibertec). The micropipette tip was left in place for 5 min before removing it from the brain. Finally, the bone defect was closed, the scalp sutured, and animals were returned to their cages.

Following 58–60 h survival, animals were overdosed with pentobarbital and perfused with saline (1 min), followed by 4% PFA in 0.1 m PB, pH 7.4, for 8 min. Their brains were then removed, immersed overnight in the same fixative at 4°C and cryoprotected by soaking in a sucrose solution (30% in PB) for further 24 h. Serial 50-μm-thick coronal sections were cut on freezing microtome (Leica Microsystems).

To allow intensive high-magnification microscopic analysis, labeling was made stable and opaque by using immunohistochemistry against eGFP and glucose oxidase-nickel enhancement (Shu et al., 1988). Free-floating sections were incubated in rabbit anti-GFP serum (1:500; EXBIO), followed by incubation with a biotinylated goat anti-rabbit serum (1:100; Sigma-Aldrich) and an avidin-biotin-peroxidase kit (1:100; Vectastain Elite, Vector Laboratories). Sections were then serially mounted onto gelatin-coated glass slides, air-dried, lightly counterstained with thionin, dehydrated, and coverslipped with DePeX (Serva Electrophoresis).

Sections were examined under bright-field optics at 10–40×. Labeled axons of Po neurons were found to span >30 serial coronal sections. Complete 2D-reconstructions of each axon were drawn using a Nikon camera Lucida, scanned, and redrawn using a vector graphics software (Canvas X, ACD Systems).

Under bright-field optics, axonal branches appeared as sharply labeled filaments with frequent varicose swellings. To estimate and compare the size of varicosities (putative synaptic boutons), the maximal projection area was measured from live images using a Nikon DMX1200 camera fitted to the microscope and the NIS-Elements imaging software tools (v3.2; Nikon). To this end, the major perimeter of each varicosity was focused at 1000× and delineated over the computer screen using the “polyline” and “polygon” software tools. For each cortical area, layer and axon, 50 randomly selected varicosities were measured. Varicosities with cross-sectional areas (maximal projection) near or below the microscope resolution limit (<0.2 μm^2^) we not included.

##### Experimental design and statistical analysis.

EM analysis was performed on samples taken from four adult male wild-type mice. These animals had received a stereotaxic BDA iontophoretic microinjection in the thalamus that, upon light microscopy assessment of a series of sections parallel to that used for EM, was found to be completely confined to the Po nucleus (Fig. 1; Table 1-1). In each experiment, the cortical regions selected for EM analysis were those containing the heaviest axonal labeling (Fig. 2).

The single-axon analysis was performed in animals that had each an individual Po cell electroporated with Sindbis-Pal-eGFP RNA. Of a larger sample of individually labeled and fully reconstructed Po cells (*n* = 14; data not shown) every cell whose axon was found to target the vibrissal domain of the motor cortex (*n* = 3) were analyzed for this study.

Statistical analysis was computed using SPSS Statistics software v24 (IBM). The threshold level of significance was set at *p < 0.05, **p < 0.01, and ***p < 0.001.

For each ultrastructural parameter, a mean ± SD, the median, maximum, and minimum values and a coefficient of variation (CV) were calculated. For subsequent data analysis for multiple comparisons we used one-way ANOVA plus T3 Dunnett's as a *post hoc* test, or Fisher's exact test.

All synaptic boutons reconstructed were clustered according to six structural parameters investigated, namely: bouton volume, bouton surface area, number of PSDs/bouton, number of mitochondria/bouton, volume of mitochondria, and PSD surface area. The cluster analysis was performed using MATLAB and Statistics Toolbox Release 2016b (MathWorks). The aim was to identify the synaptic parameters that best characterized the synaptic boutons investigated. Thus, a principal component analysis was performed to simplify the dataset and to convert a set of observations of possibly correlated variables into a set of values of linearly uncorrelated variables called principal components (PCs; Abdi and Williams, 2010). Then, we performed a hierarchical cluster analysis (Rokach and Maimon, 2005) on the new simplified dataset composed of the PCs. This method is used for unsupervised machine learning when the original data were unlabeled (Yakoubi et al., 2019).

For single-cell labeling experiments, comparison of mean sizes of varicosities (maximal projection 2D areas) between different cortical areas/layers was performed using Mann–Whitney *U* test. Two-sample Kolmogorov–Smirnov test (K–S) was used to compare size distributions of varicosities between areas/layers.

## Results

### Light microscopic visualization of Po axon terminals in S1 and in MC

To selectively label a sizable population of thalamocortical Po axons, 10 kDa BDA was iontophoretically delivered into Po. Only experiments in which the BDA deposit was restricted to the Po nucleus were analyzed (Fig. 1). In S1, two distinct bands of axonal arborizations were labeled: one in L5a and the other in upper half of L1 (Fig. 1*c*). In MC, labeled Po axonal arborizations formed a single band from upper L5a to lower L3 (Fig. 1*d*). The Golgi-like axon staining revealed frequent varicosities of variable size (Fig. 1*e–g*).

### ssTEM and FIB-SEM analysis of labeled thalamocortical Po axons

EM samples were taken from sections adjacent to those containing the heaviest anterograde labeling in S1 or MC. These were subsequently analyzed using either high-resolution, fine-scale ssTEM or FIB-SEM. In S1, samples were taken from L1 and L5a, which were readily delineated cytoarchitecturally. However, the cytoarchitectonic definition of MC middle layers is ambiguous; thus, our MC samples included L4 and adjacent deep parts of L3; we refer to this neuropil as “MC-L4/3”.

**Figure 3.**
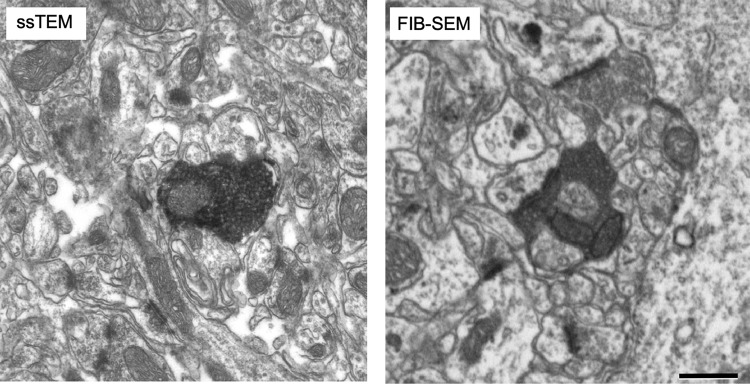
Image resolution of ssTEM versus FIB-SEM. Representative electron micrographs comparing the visualization of tissue details obtained with ssTEM (left) or FIB-SEM (right) at the scanning resolutions applied in this study. Scale bar, 0.5 μm.

The two serial 3D EM techniques yielded in consistent results. The ssTEM analysis allowed a high-resolution 3D-volume reconstruction of the overall geometry of synaptic complexes including the subsequent quantification of PSD surface area and vesicle pool size; however, it sampled only axon varicosities, and a smaller neuropil volume, which not always allowed a full visualization of the spine neck. In turn, FIB-SEM analysis allowed the complete visualization of postsynaptic elements and inter-bouton axonal segments. For efficiency, the FIB-SEM image acquisition was made with a slightly lower resolution than the ssTEM images; for this reason, fine details such as the individual synaptic vesicles could be only unambiguously resolved and counted in the ssTEM samples (Fig. 3).

### Ultrastructural features of Po axons in the vibrissal regions of S1 and MC

In the following account, we use the terms *bouton* and *varicosity* interchangeably to describe axonal swellings of >0.5 μm of diameter or exceeding by >50% the typical variation of the adjacent axonal segments (Shepherd and Harris, 1998). The majority of such boutons/varicosities (93–98%) were synaptic sites (i.e., were apposed to at least one PSD). Most of them (88–92%) contained also at least one mitochondrion (Tables 1, 2). A small number of boutons, however, were nonsynaptic; these were essentially a mitochondrion, usually with some presynaptic vesicles, situated in a swollen axon region. Nonsynaptic varicosities are indicated by asterisks in Figures 5 and 6.

**Table 1.** Ultrastructural 3D measurements of Po synapses in MC-L4/3, S1-L1 or S1-L5a, and comparison with VPM synapses in S1-L4: presynaptic element

	Non-bouton synapses, %*	Synapses/μm axon length*	No. of boutons analyzed	Synapses/bouton	Bouton surface area, μm^2^		Bouton volume, μm^3^		Multisynaptic boutons, %	Nonsynaptic mitochondria, %*	Boutons containing mitochondria, %	Mitochondrial volume/bouton, μm^3^		No. of synaptic vesicles/bouton**	
S1 L5a	28.5	0.17	74	1.1	Range	0.86 – 6.84	Range	0.03 – 0.8	9	7	88	Range	01 – 0.19	Range (N = 12)	142 – 878
	(6/21)				Mean ± SD	2.69 ± 1.15	Mean ± SD	0.24 ± 0.13				Mean ± SD	0.06 ± 0.04	Mean ± SD	346 ± 211
					Median	2.52	Median	0.23				Median	0.06	Median	300
					CV	0.43	CV	0.54				CV	0.6	CV	0.61
S1 L1	25	0.05	67	1.1	Range	1.34 – 7.09	Range	0.01 – 0.89	12	7	90	Range	0.02 – 0.16	Range (N = 10)	394 – 1156
	(1/4)				Mean ± SD	3.58 ± 1.43	Mean ± SD	0.37 ± 0.21				Mean ± SD	0.06 ± 0.03	Mean ± SD	733 ± 294
					Median	3.67	Median	0.34				Median	0.05	Median	661
					CV	0.4	CV	0.56				CV	0.56	CV	0.4
MC L3/4	23	0.14	51	1.4	Range	1.35 – 11.3	Range	0.09 – 1.06	29	2	92	Range	0.02 – 0.23	Range (N = 7)	310 – 1029
	(3/13)				Mean ± SD	4.3 ± 2.15	Mean ± SD	0.39 ± 0.22				Mean ± SD	0.08 ± 0.04	Mean ± SD	613 ± 257
					Median	3.72	Median	0.35				Median	0.07	Median	565
					CV	0.5	CV	0.56				CV	0.55	CV	0.42
S1 L4 (VPM)	5.3	0.34	76	1.6	Range	1.26 – 11.58	Range	0.06 – 1.59	53	0	92	Range	0.01 – 0.25	Range (N = 12)	200 – 1095
	(3/56)				Mean ± SD	4.67 ± 2.20	Mean ± SD	0.46 ± 0.27				Mean ± SD	0.09 ± 0.06	Mean ± SD	740 ± 285
					Median	4.42	Median	0.42				Median	0.09	Median	795
					CV	0.47	CV	0.59				CV	0.58	CV	0.39

*FIB-SEM data only.

**ssTEM data only.

All other are pooled ssTEM and FIB-SEM data. See extended Table 1-1 for details of the cases and areas/layers sampled.

10.1523/JNEUROSCI.2886-19.2020.t1-1 Table 1-1 Detail of the experimental cases used for the EM analysis, tissue samples examined and the 3D EM techniques applied on them. Download Table 1-1, DOCX file

**Table 2.** Ultrastructural 3D measurements of Po synapses in MC-L4/3, S1-L1 or S1-L5a, and comparison with VPM synapses in S1-L4: postsynaptic element

	No. of analyzed synapses	Synapses on spines vs dendritic shafts, %	PSD shape, %		PSD surface area, μm^2^		Synapses per mitochondrion	No. of analyzed spines	Spine head volume, μm^3^		Spines containing spine apparatus, %	Spines with a protrusion embedded into the bouton, %	Spines with an additional (symmetric) synapse, %
S1 L5a	80	83 vs 0.17	Disc	65	Range	0.01 – 0.25	1.05	66	Range	0.01 – 0.19	27	6	18
			Horseshoe	16	Mean ± SD	0.11 ± 0.06			Mean ± SD	0.06 ± 0.03			
			Perforated	10	Median	0.1			Median	0.05			
			Fragmented	9	CV	0.52			CV	0.6			
S1 L1	71	96 vs 4	Disc	43	Range	0.02 – 0.51	0.9	68	Range	0.01 – 0.28	31	3	3
			Horseshoe	37	Mean ± SD	0.16 ± 0.1			Mean ± SD	0.11 ± 0.06			
			Perforated	17	Median	0.14			Median	0.1			
			Fragmented	3	CV	0.6			CV	0.58			
M1 L3/4	69	94 vs 6	Disc	41	Range	0.03 – 0.41	1.15	65	Range	0.01 – 0.2	69	19	9
			Horseshoe	29	Mean ± SD	0.17 ± 0.08			Mean ± SD	0.09 ± 0.05			
			Perforated	22	Median	0.15			Median	0.09			
			Fragmented	8	CV	0.5			CV	0.51			
S1 L4 (VPM)	124	83 vs 0.17	Disc	49	Range	0.01 – 0.24	1.4	92	Range	0.004 – 0.18	75	13	15
			Horseshoe	35	Mean ± SD	0.1 ± 0.05			Mean ± SD	0.06 ± 0.04			
			Perforated	9	Median	0.09			Median	0.06			
			Fragmented	7	CV	0.5			CV	0.62			

A total of 192 axon boutons were reconstructed and analyzed with ssTEM or FIB-SEM: 74 BDA-labeled boutons from S1-L5a, 67 boutons from S1-L1, and 51 boutons from MC-L4/3 (Tables 1, 2). Labeled boutons were identified by their electron-dense DAB reaction product in their cytoplasm. The sampling of labeled boutons in ssTEM was essentially random, as any labeled swollen axon region (>0.5 μm diameter) that could be followed from its beginning to its end within a given series of ultrathin sections was reconstructed and quantified. In FIB-SEM image stacks, all the labeled axonal segments contained in the stack volume, varicose or not, were analyzed.

A total of 220 Po synapses established by labeled Po axons and their respective target structures were reconstructed and analyzed with ssTEM or FIB-SEM: 80 in S1-L5a, 71 in S1-L1, and 69 in MC-L4/3. Synaptic contacts were identified by the presence of distinct, parallel presynaptic and postsynaptic membranes at the synaptic apposition zone, separated by a synaptic cleft and an electron-dense band adherent to the cytoplasmic surface of the postsynaptic membrane (PSD). This corresponds to asymmetric synapses, regarded as excitatory and glutamatergic (Peters and Palay, 1996; Ascoli et al., 2008).

**Figure 4.**
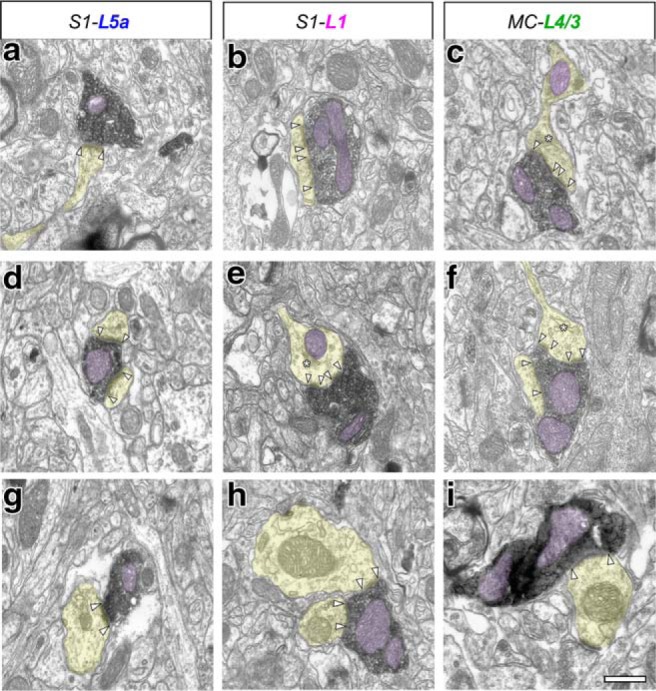
Electron micrograph of BDA-labeled Po thalamocortical boutons establishing synaptic contacts with cortical dendritic spines or shafts. In all images, dendritic shafts and spines are highlighted in transparent yellow and mitochondria are shaded in transparent light purple. PSD borders are marked by white arrowheads. Asterisks indicate the spine apparatus. Inside some boutons, synaptic vesicles are clearly visible. ***a***, ***d***, Typical Po boutons synapsing on a dendritic spine head (***a***) or simultaneously onto two different spine heads (***d***) in S1–L5a. ***b***, ***e***, Po boutons synapsing on dendritic spines in S1–L1. ***e***, Note the presence of a mitochondrion and a spine apparatus (asterisk) within the postsynaptic spine head. ***c***, ***f***, Synaptic Po boutons in MC-L4/3 synapsing onto one (***c***) or two dendritic spines simultaneously (***f***). ***g–i***, Examples of Po bouton terminating onto dendritic shafts, one in each of the three studied neuropils. Scale bar, 0.5 μm.

In the neuropil of the three regions studied, the large majority of Po axon boutons (70–90%) were monosynaptic. Note, in addition, that the FIB-SEM analysis indicates the presence of numerous (∼25%) synapses in the non-varicose inter-bouton segments, which were always monosynaptic. Thus, the actual proportion of total monosynaptic sites in Po axons may be even higher (∼77–96%). Approximately 30% of the boutons in MC-L4/3 and ∼10% in S1 (L5a and L1) simultaneously contacted two different postsynaptic structures (Fig. 4*d*,*f*; Tables 1, 2). We did not observe any Po bouton with three or more synapses, which is a frequent finding in S1-L4 VPM boutons (Tables 1, 2). Non-varicose synaptic sites are rare (∼5%) in VPM axons (Rodriguez-Moreno et al., 2018).

**Figure 5.**
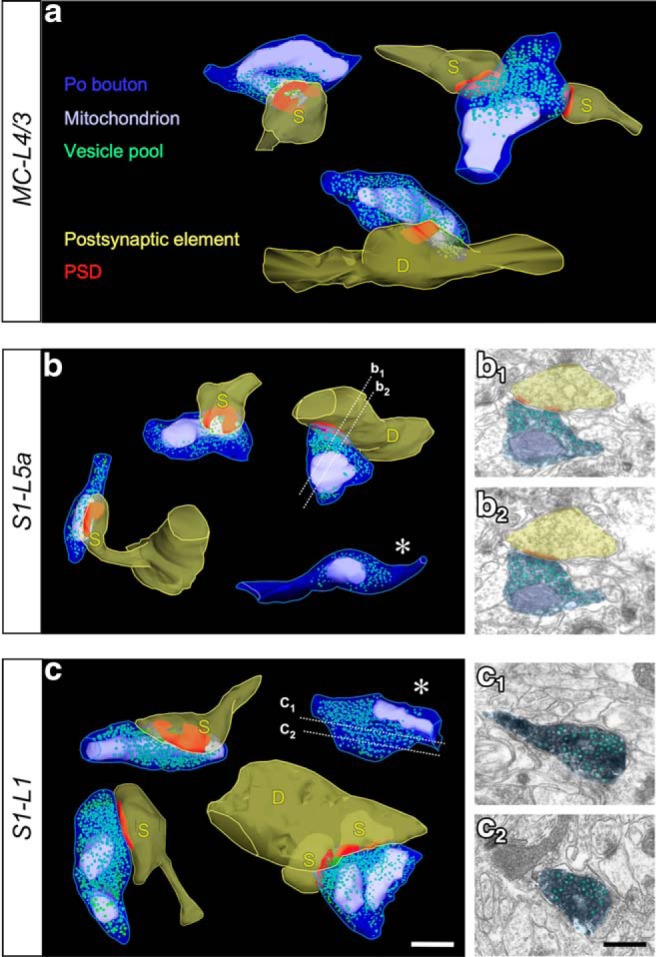
ssTEM 3D-reconstructions of thalamocortical Po boutons in the three cortical domains investigated. ***a***, Po Boutons labeled in MC-L4/3. Top, Two boutons contacting spines (S). The bouton on the right is simultaneously establishing synaptic contacts with two different spines. Bottom, Synaptic bouton terminating on a dendrite shaft (D). (***b***) Po Boutons in S1–L5a. Left, Two boutons contacting dendritic spines. Right, top, Bouton synapsing on a dendritic shaft (D). Two of the serial sections (indicated by dashed white lines) of which this bouton reconstruction was created are illustrated in ***b_1_*** and ***b_2_***. Right, bottom (white asterisk), Small axon varicosity containing a mitochondrion and synaptic vesicles, but no adjacent PSD. ***c***, Po Boutons labeled in S1–L1. Left, Two boutons establishing synaptic contacts with mushroom-like dendritic spines. Right, top, (white asterisk), Large axonal varicosity containing a mitochondrion and synaptic vesicles, but no adjacent PSD. Two of the sections from which this reconstruction was created are illustrated in ***c_1_*** and ***c_2_***. Right, bottom, Bouton simultaneously contacting a dendritic shaft and two different spines. Scale bar, 0.5 μm.

Most Po varicosities (88–92%; Tables 1, 2) contained one or several mitochondria of different shape and size (Figs. 4, 5). Mitochondria contributed substantially to the bouton volume (25% in S1-L5a, 16% in S1-L1, and 20.5% in MC-L4/3). Synaptic vesicles were clearly visible and, in the boutons less heavily-stained (*n* = 12 in S1-L5, 10 in S1-L1, and 7 in MC-L4/3) it was even possible to identify and count all the synaptic vesicles.

In both S1-L1 and S1-L5a, a significant number (7%) of Po axonal varicosities contained a mitochondrion and some synaptic vesicles, yet lacked any evident synaptic contact (Fig. 5*b*,*c*). Such nonsynaptic boutons were also observed in MC-L4/3 Po axons although less frequently (2%). Such boutons are virtually absent in the VPM S1-L4 axons (Tables 1, 2; White et al., 2004).

### Synaptic structure features specifically revealed by FIB-SEM analysis

Our FIB-SEM analysis is based on sampling 2435 μm^3^ of cortical neuropil. At the magnification used, the axonal BDA axonal segments were few and widely scattered. Thus, despite taking our samples from zones that under light microscopy appeared heavily labeled (Fig. 1), many of the FIB-SEM image stacks actually contained few or no labeled Po axonal segments.

**Figure 6.**
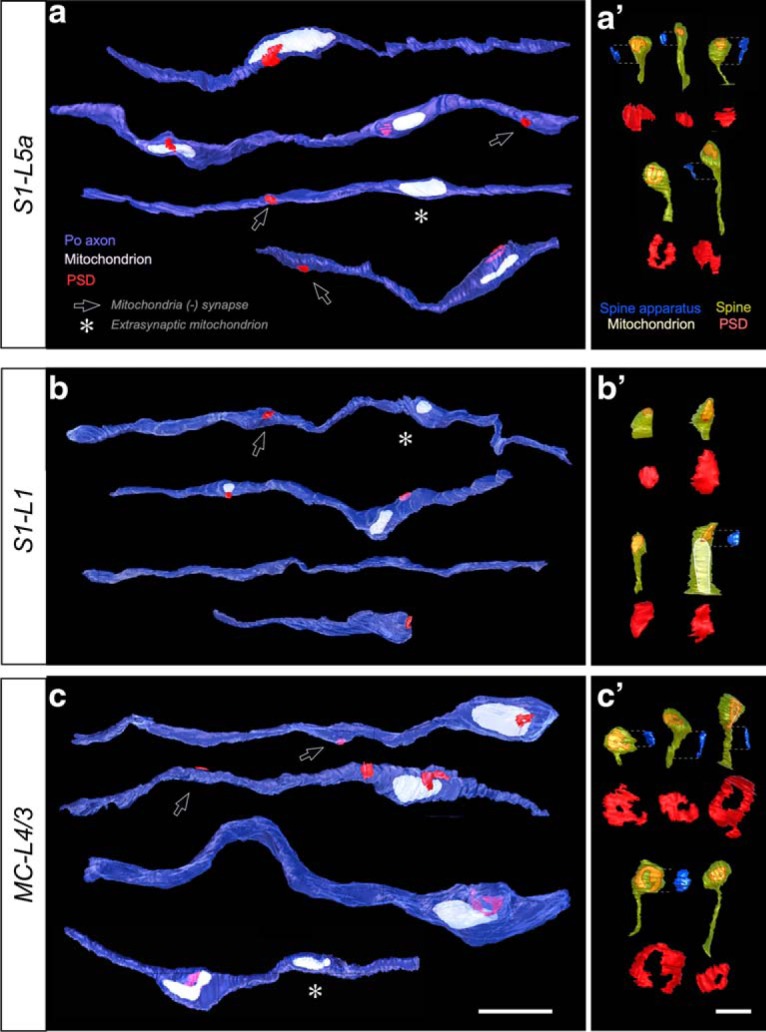
Representative examples of thalamocortical Po axon segments, postsynaptic dendritic spines and PSDs 3D-reconstructed from FIB-SEM image stacks. ***a***–***c***, Individual axonal segments from (***a***) S1–L5a; (***b***) S1–L1, or (***c***) MC-L4/3. Black arrows indicate PSDs located in non-varicose axon domains. Asterisks indicate mitochondria not associated to a PSD (“nonsynaptic” mitochondria). Scale bar, 2 μm. ***a′***–***c′***, Different morphologies of dendritic spines postsynaptic to Po boutons in (***a′***) S1–L5a, (***b′***) S1–L1, and (***c′***) MC-L4/3. Spine surface is partly transparent to allow the visualization of the PSDs within the spine head. In the spines containing a spine apparatus, this organelle (blue) is shown aside. Scale bars (for spines and spine apparati): 0.5 μm. For clarity, the spine PSDs are also represented isolated below, at double magnification. Note the differences in both shape (perforated vs non-perforated, closed ring- or horseshoe-like) and size of the PSDs. ***b′***), Note the large spine containing a mitochondrion.

**Figure 7.**
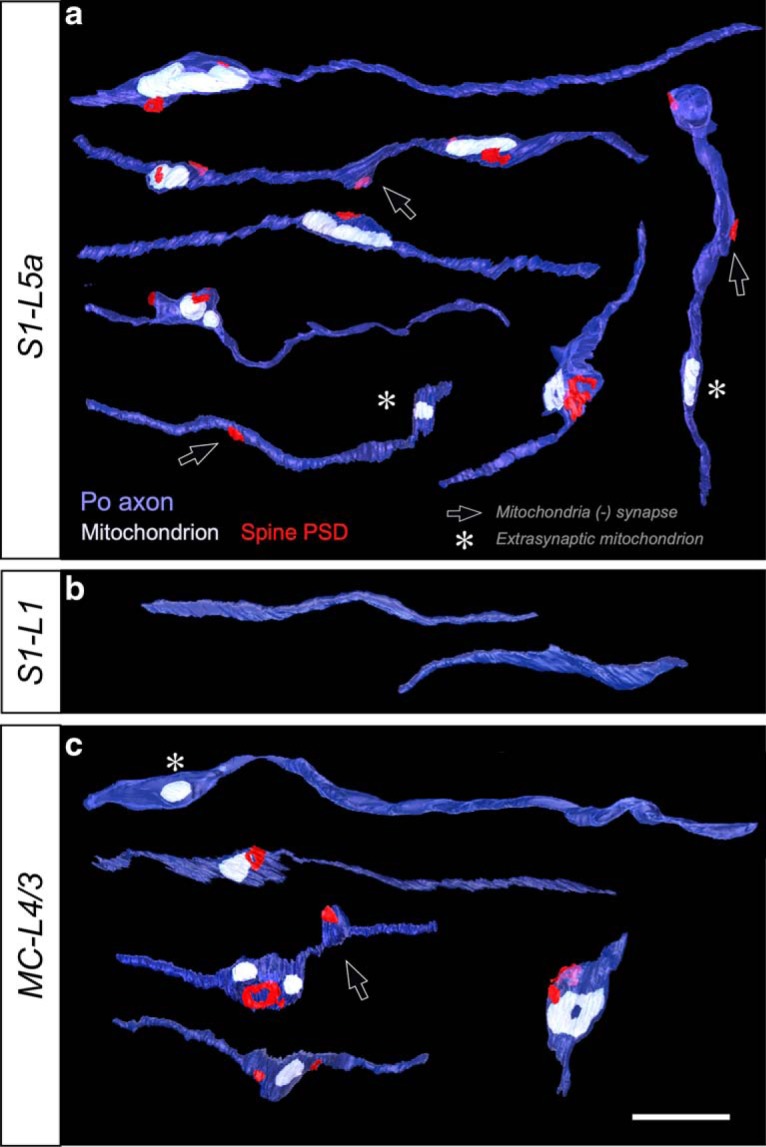
Additional thalamocortical Po axonal segments 3D-reconstructed from FIB-SEM image stacks. ***a***–***c***, Individual axonal segments from (***a***) S1–L5a, (***b***) S1–L1, or (***c***) MC-L4/3. All the PSDs in these particular segments were located on spines. Black arrows highlight PSDs located in non-varicose axonal domains. Scale bar, 2 μm.

Nevertheless, 26 Po axonal segments totaling ∼283 μm of axonal length were reconstructed (Tables 1, 2; Table 1-1). Of these, 108 μm were measured from axonal branches in S1-L5a (0.16 synapses/μm total axon length); 74 μm from S1-L1 (0.05 synapses/μm total axon length); and 101 μm from MC-L4/3 (0.14 synapses/μm total axon length; Tables 1, 2). The morphological parameters of the boutons observed with FIB-SEM were fully consistent with those observed with ssTEM. Likewise, some varicosities contained a mitochondrion but were not apposed to a PSD (Figs. 6, 7, white asterisks). In addition, the complete visualization of long inter-bouton axonal segments provided by FIB-SEM revealed that ∼one-quarter (9 of 37) of synapses occur in these segments (Tables 1, 2; Figs. 6*a–c*, 7). Synapses in non-varicose segments are rarer in VPM S1-L4 axons (White et al., 2004; Rodriguez-Moreno et al., 2018).

### Ultrastructural features of elements postsynaptic to the Po axons in MC and S1

In all three cortical regions investigated, the majority of Po synapses (83–96%) were established on spines. Synapses onto dendritic shafts (which may partially correspond to non-spiny cortical interneurons; Bopp et al., 2017) were frequent (17%) in S1-L5a, but less frequent by ∼fourfold in S1-L1 or MC-L4/3 (4–6%, respectively; Tables 1, 2; Figs. 4–6). No contacts on neuronal somata were observed.

Strikingly, the PSDs displayed a wide range in both shape and size (Tables 1, 2; Fig. 6*a′–c′*). The mean surface area of the S1-L5a PSDs was similar (0.11 μm^2^) to that previously measured in VPM-L4 synapses (Tables 1, 2; Fig. 9*e*). In contrast, the mean PSDs surface area of MC-L4/3 and S1-L1 Po synapses were ∼60% larger. In S1-L5a, most PSDs (65%) had disc-like morphologies, whereas most of those in MC-L4/3 (59%) displayed complex horseshoe-shaped, perforated, or fragmented morphologies (Fig. 6*a′–c′*).

A total of 199 spines postsynaptic to Po boutons were analyzed (Tables 1, 2). Some of them (9% in MC-L4/3, 3% S1-L1, and 18% in S1-L5) were also targeted by an unlabeled symmetric (putatively inhibitory) synapse of unknown origin. Although only 30% of the spines postsynaptic to Po boutons in S1 (L5a and L1) contained a spine apparatus (an endoplasmic reticulum derivate, Fig. 6*a′*,*b′*), the large majority (70%) of postsynaptic spines in MC-L4/3 displayed this structural sub-element responsible for spine motility but also stabilization of the presynaptic and postsynaptic apposition zone during synaptic transmission. Mitochondria were found inside 2 of the 68 spines postsynaptic to S1-L1 Po boutons, despite the exceedingly rare occurrence of spine mitochondria in rodent somatosensory cortex (Santuy et al., 2018).

### Dendritic spine protrusions into the thalamocortical Po boutons

**Figure 8.**
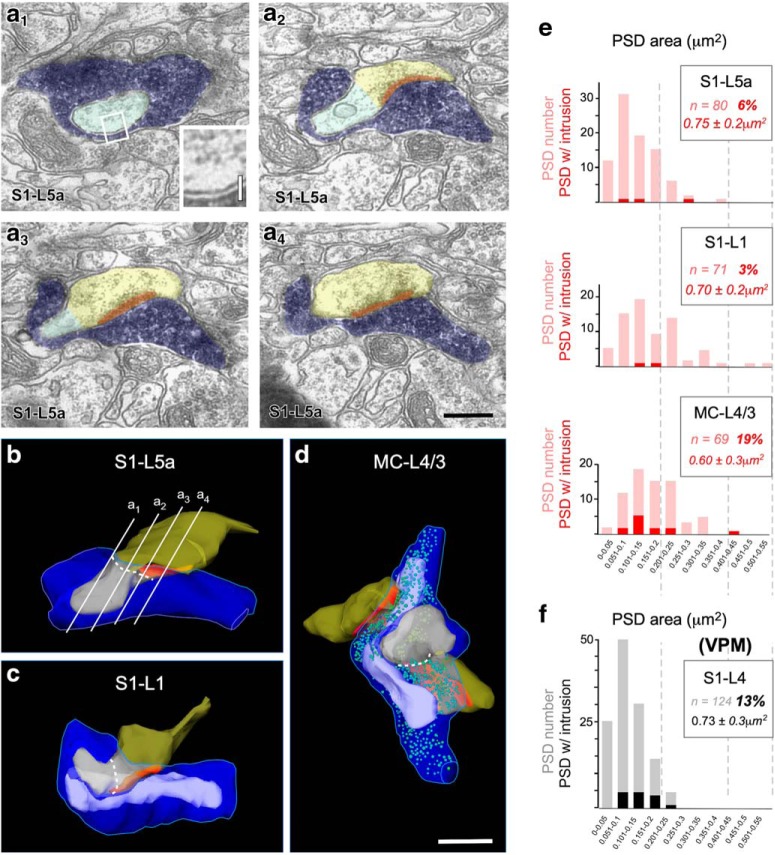
Large cortical dendritic spine protrusions invaginate into the presynaptic Po boutons. ***a_1_***–***a_4_***, Consecutive electron micrographs showing a postsynaptic spine head (transparent yellow) with a large protrusion (transparent green) invaginated into a labeled Po bouton (transparent blue). Note that the PSD (red) is adjacent to the protrusion, but not extending into it. Note also the close apposition of the presynaptic and postsynaptic cell membranes in the intruded region (***a_1_***, inset). Scale bars: ***a_1_***–***a_4_***, 0.5 μm; ***a_1_***, inset, 0.1 ***b***–***d***, 3D ssTEM reconstructed examples of synaptic boutons in which a cortical spine protrusion is invaginated into a labeled presynaptic Po bouton. A dashed white line highlights the approximate border between the spine protrusion and the remaining spine head. The synaptic bouton in ***b*** is a reconstruction made from the same series of sections shown in ***a_1_***–***a_4_*** (red lines). The reconstructions in ***b*** and ***c*** are shown without synaptic vesicles because the BDA-DAB labeling made vesicle delineation unreliable in these two particular boutons. Scale bar, 0.5 μm. ***e***, Bar histograms showing the distribution in PSD surface area in thalamocortical Po synaptic boutons in each of the three cortical regions examined. Total numbers of PSD are represented in pink. PSDs with a dendritic spine protrusion adjacent to them are highlighted in red. In the framed area, the total number of PSD measured in each cortical region, the percentage of PSDs with adjacent protrusions, and the mean surface area ± SD of the protrusion membrane are indicated. ***f***, To allow a direct comparison, PSD surface areas and protrusions measured in S1–L4 VPM synapses (Rodriguez-Moreno et al., 2018) are displayed in gray/black.

Numerous dendritic spine heads postsynaptic to Po boutons formed a thick finger-like protrusion embedded into the presynaptic bouton (Fig. 8*a–d*). Such invaginations were observed in all three cortical domains investigated, but were substantially more frequent in MC-L4 than in the S1 Po synapses (Fig. 8*e*; Tables 1, 2). Their volume was similar in the three cortical domains analyzed (range 0.6–0.75 μm^3^). We had observed similar invaginations in mouse S1-L4 VPM synapses; with a prevalence similar to that in Po MC synapses (13 vs 19%; Fig. 8*e*,*f*; Rodriguez-Moreno et al., 2018). Previous 2D EM studies in ferret (Erisir and Dreusicke, 2005) and tree shrew visual cortices (Familtsev et al., 2016) reported even more frequent protrusions in geniculo-cortical axons. Our observations thus suggest that spine protrusions may be a common frequent feature of mammalian thalamocortical synapses on spiny stellate and pyramidal cells.

Protrusions were observed in spines with widely different PSDs sizes (Fig. 8*e*) although were not found near the smallest (< 0.05 μm^2^; Fig. 8*e*,*f*). Remarkably, the spine PSD was always adjacent to, but outside the protrusion's edge (the point where the protrusion membrane became apposed to that of the thalamocortical bouton) and we never observe a PSD inside a protrusion. The invaginated intermembrane surface was large (up to 20 times the size of the active zone). The invaginated membranes were smooth, remained closely apposed (∼20–40 nm) and lacked evident membrane specializations (Fig. 8*a*_1_). Diffusion of secreted molecules in such an extensive, narrow and isolated intermembrane space is probably nonlinear and free of direct glial scavenging. In addition to, is it even possible that the extensive parallel, tightly apposed membrane surfaces may allow local electric field (ephaptic) conduction (Petralia et al., 2018) between the postsynaptic spine and the thalamocortical bouton.

### Quantitative comparisons of structural synaptic parameters

**Figure 9.**
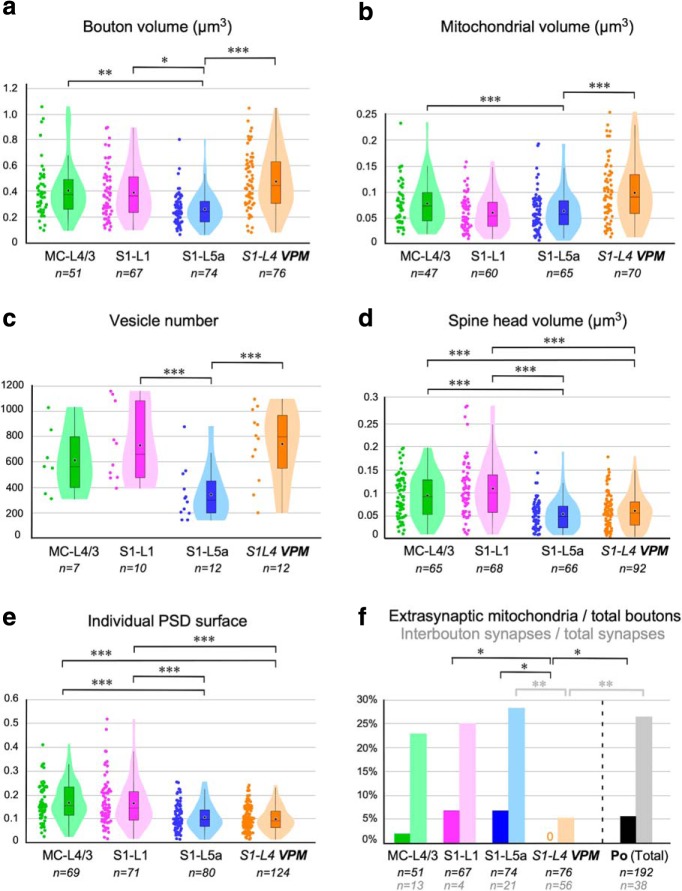
Structural parameters distributions and paired comparisons between MC-L4/3, S1–L1, S1–L5a Po synapses and S1–L4 VPM synapses. ***a–c***, Structural parameters of the presynaptic (thalamocortical) element: (***a***) bouton volume; (***b***) mitochondrial volume; and (***c***) synaptic vesicle number displayed both as dot plots and violin charts. Violin charts show density distribution of the data. Superimposed box plots show median (black line), mean (black dot) and interquartile range. Whiskers extend to lowest and highest non-outlier values. ***a***, ***b***, Shows ssTEM + FIB-SEM data pooled together; (**c**) shows only ssTEM data. ***d***, ***e***, Postsynaptic spine head volumes (***d***) and postsynaptic density interface areas (spine + shaft synapses). Both panels show pooled ssTEM + FIB-SEM data. ***f***, Comparison of ratios of mitochondria not associated with an active zone over total boutons (FIB-SEM data) and postsynaptic densities in inter-bouton segments not associated (>2 μm away) with mitochondria over total PSDs (FIB-SEM data). No extrasynaptic mitochondria were found in VPM axons and thus the value is indicated as “0”. Levels of significance are indicated by asterisks: *p < 0.05, **p < 0.01, ***p < 0.001.

To substantiate the above observations, structural parameters of Po axon synapses were statistically compared between in the three cortical domains investigated (one-way ANOVA, Dunnett's *post hoc* test; Fig. 9; Tables 1, 2). This analysis revealed several striking differences. For example, Po boutons in MC-L4/3 were significantly larger (∼60%) in volume than those in S1-L5a (0.39 ± 0.22 vs 0.24 ± 0.13 μm^3^, *p* = 0.003; Fig. 9*a*). Mitochondrial volume per bouton was ∼33% larger in MC-L4/3 versus S1-L5a Po boutons (0.08 ± 0.04 vs 0.06 ± 0.04 μm^3^, *p* < 0.001; Fig. 9*b*). Vesicle pools in both MC-L4/3 and S1-L1 boutons were, as a mean, approximately twice as large as those in S1-L5a boutons. Because of the limited number vesicle pools in our MC sample, the statistical significance could be confirmed for S1-L1 versus S1-L5a Po boutons (733 ± 294 vs 346 ± 211, *p* = 0.040; Tables 1, 2; Fig. 9*c*). In addition, both the head volume and the PSD surface area of the spines postsynaptic to Po boutons in MC-L4/3 (0.09 ± 0.03 μm^3^, 0.17 ± 0.08 μm^2^) and S1-L1 (0.11 ± 0.06 μm^3^, 0.16 ± 0.08 μm^2^) were significantly larger (+50%) than those of spines postsynaptic to S1-L5a Po boutons (0.06 ± 0.03 μm^3^, 0.11 ± 0.06 μm^2^; *p* < 0.001 in all comparisons, except S1-L1 vs L5a PSD surface: *p* = 0.003; Fig. 9*d*,*e*).

Next, we compared the structure of synaptic boutons established by Po versus VPM thalamic nuclei axons in S1 by putting side by side the 3D Po bouton measurements and those of VPM S1-L4 boutons (Rodriguez-Moreno et al., 2018; Tables 1, 2). This comparison revealed that the VPM-L4 boutons are ∼90% larger in volume (0.46 ± 0.27 μm^3^, *p* < 0.001), and ∼50% in mitochondrial volume (0.09 ± 0.06 μm^3^, *p* < 0.001) and contained more than twice synaptic vesicles (740 ± 285, *p* = 0.010) than Po-L5a boutons (Tables 1, 2; Fig. 9*a–c*). In contrast, the Po MC-L4/3 boutons are statistically indistinguishable from VPM S1-L4 boutons with respect to these three parameters (*p* > 0.05 in all cases). Overall, these comparisons reveal that axons from two different thalamic nuclei can form structurally different presynaptic specializations in adjacent layers of the same cortical columns as well as those axons from the same thalamic nucleus can form structurally different specializations in separate areas/layers.

The S1 spines postsynaptic to both VPM axons (in L4) and Po axons (in L5a) have almost identical head volumes and PSD sizes (0.06 ± 0.04 μm^3^ and 0.10 ± 0.05 μm^2^; *p* > 0.05 in both cases). In contrast, spines postsynaptic to Po axons were much larger in S1-L1 (83 and 45%, respectively) or MC-L4/3 (50 and 54%; *p* < 0.001 in all cases; Fig. 9*d*,*e*), consistent with the notion that postsynaptic element differences reflect to a larger extent the specific cell types and/or dendritic domains present in separate cortical layers or areas.

In addition to synaptic boutons, we also compared the frequencies of extra-synaptic mitochondria (those found in axonal varicosities without an active zone; Fig. 9*f*). The differences were found to be statistically significant (Fisher's exact test) between VPM-L4 axons and Po axons as a whole (0 vs 5.7%, *F* = 0.037, *p* < 0.05), as well as between VPM-L4 axons and Po axons in S1-L1 (0 vs 7.4%, *F* = 0.021, *p* < 0.05) or S1L5a (0 vs 6.7%, *F* = 0.027, *p* < 0.05). We also compared the distribution of synapses situated in inter-bouton segments, > 2 mm away from any mitochondrion (Fig. 9*f*). Again the differences were significant between the VPM-L4 axons and Po axons in MC and S1 as a whole (5.3 vs 26.3%, *F* = 0.005, *p* < 0.01), as well as between VPM-L4 axons and Po-L5a axons in S1 (5.3 vs 28.7%, *F* = 0.01, *p* < 0.05). Together, the above results indicate that the axon mitochondria of Po axons in S1 and MC are far less bound to synaptic sites than in VPM S1-L4 axons.

**Figure 10.**
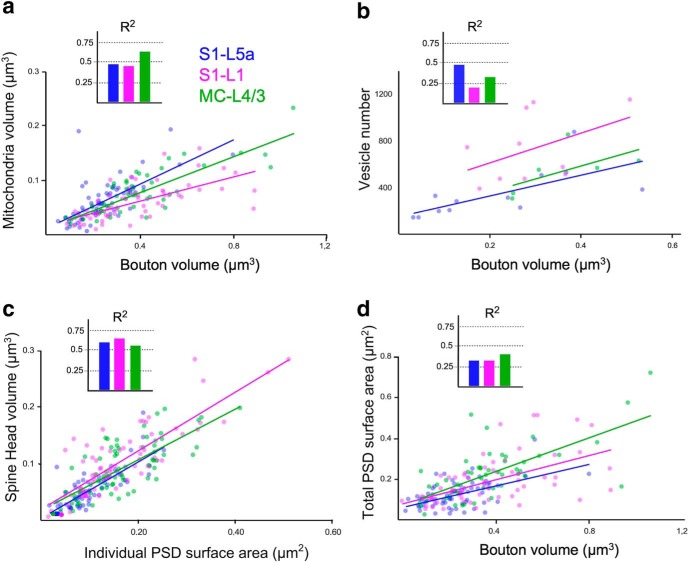
Correlation analyses between structural parameters of Po synaptic boutons in the three cortical regions analyzed. ***a***–***d***, Dot plots showing the correlations between: (***a***) bouton volume versus mitochondrial volume, (***b***) bouton volume versus number of synaptic vesicles, (***c***) Individual PSD surface area versus spine head volume, (***d***) bouton volume versus total PSD surface area. Correlations are indicated by linear regression as well as by the coefficient of correlation (*R*^2^). These are compared, for clarity, as bar histograms.

**Figure 11.**
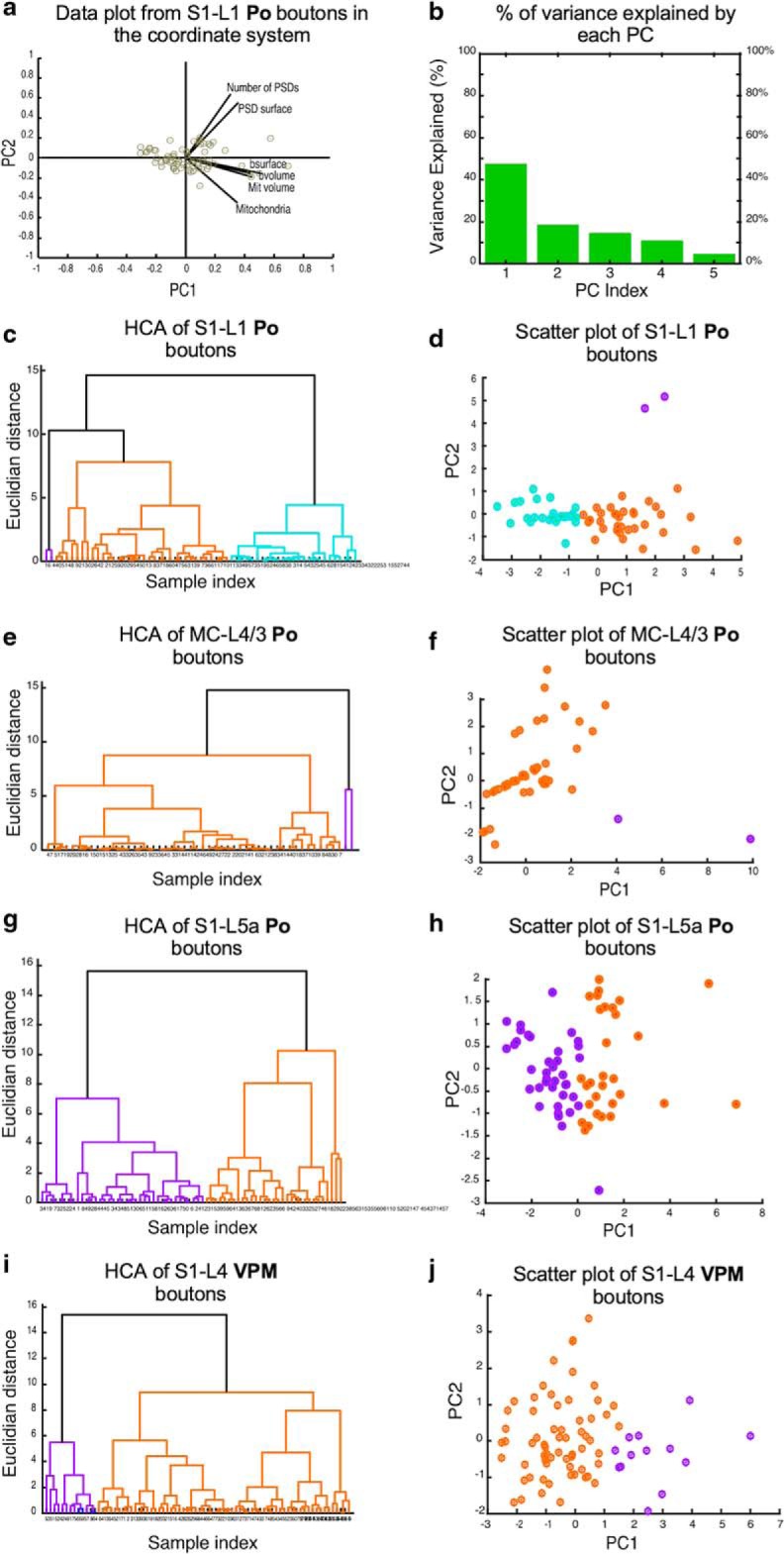
Cluster Analysis comparisons of the various Po synapse parameters. ***a***, Projection of the original data of S1–L1 Po synaptic boutons in the new coordinate system based on the PCs, showing the coefficients for each variable and scores for each observation. bvolume, bouton volume; bsurface, bouton surface area; Mit *volume*, mitochondrial volume. ***b***, Histogram of the percentage of variance explained by the PCs for data from S1–L1 Po synaptic boutons. The PCs considered as main PCs are 1 and 2 as their total explain variance is ∼70%. ***c***–***j***, Dendrogram and scatter plot analyses of Po synaptic bouton parameters in each of the cortical regions examined. ***c***, ***d***, Data plots for S1–L1. ***e***, ***f***, Data plots for MC-L4/3. ***g***, ***h***, Data plots for S1–L5a. In addition, the same type of data plots for VPM boutons in S1–L4 (Rodriguez-Moreno et al., 2018) is shown in ***i*** and ***j***. In all plots, orange and purple colors are used to show the major clusters with respect to the synaptic parameters analyzed. Dissimilarity between clusters is indicated by the Euclidean distance and height.

Finally, to detect possible associations between pairs or groups of features in the structure of the Po synaptic varicosities, we conducted a correlation (*R*^2^, linear regressions; Fig. 10) and cluster (Fig. 11) cross-comparisons of the various structural parameters. Synapses in non-varicose axonal zones were not included in these tests. The comparison revealed that in all cortical areas, particularly in MC-L4/3, the volume of Po synaptic varicosities is positively correlated with that of their resident mitochondria (S1-L5a: *R*^2^ = 0.45, S1-L1: *R*^2^ = 0.43, MC-L4/3: *R*^2^ = 0.67; Fig. 10*a*), yet much less so with the size of their vesicle pool (S1-L5a: *R*^2^ = 0.47, S1-L1: *R*^2^ = 0.20, MC-L4/3: *R*^2^ = 0.37) or the total surface of the varicosity PSDs (S1-L5a: *R*^2^ = 0.30, S1-L1: *R*^2^ = 0.29, MC-L4/3: *R*^2^ = 0.43; Fig. 10*b*,*c*). In each neuropil, presynaptic and postsynaptic parameters clustered independently; for example, the total PSD area per bouton was only weakly correlated with bouton size (Fig. 10*c*), but PSD surface area and the volume of the postsynaptic spine head are strongly correlated (S1-L5a: *R*^2^ = 0.62, S1-L1: *R*^2^ = 0.69, MC-L4/3: *R*^2^ = 0.54; Figs. 10*d*, 11).

In both Po and VPM boutons the correlations for bouton size with vesicle pool and for PSD surface with bouton volume seem both to be weak (Fig. 10*b*,*c*). Although our samples were relatively small and that we did not include in this analysis the smallest synapses (in inter-bouton segments), the coefficients are in consonance with results by Rollenhagen et al. (2015) for unlabeled synapses in rat S1-L4. Much stronger correlations have been reported in unlabeled terminals presynaptic to specific apical dendritic tufts in S1-L1 (Knott et al., 2006) or CA3 axons in the hippocampus CA1 stratum radiatum (Shepherd and Harris, 1998). A relatively low correlation between PSD area and presynaptic parameters might thus be a feature of thalamocortical boutons, which may be in part related to the concentration of mitochondria at synapses and/or to the prevalence of multisynaptic boutons.

### Boutons in the MC branches of Po axons are consistently larger than those in branches of the same axons in S1

**Figure 12.**
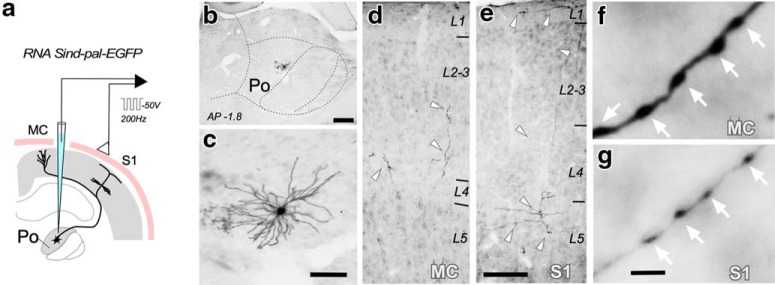
Isolated individual Po neuron labeling by means of *in vivo* electroporation of a Sindbis-pal-eGFP RNA construct. ***a***, Schematic diagram of the Sindbis-pal-eGFP RNA electroporation procedure. ***b***, Coronal section through the thalamus showing an isolated neuron transfected in Po. ***c***, Somatodendritic morphology of the neuron shown in ***b*** as seen on a single 50-μm-thick section. ***d***, ***e***, Labeled thalamocortical axonal fragments (white arrowheads) as seen in columnar ionin-counterstained samples from MC (***d***) and S1 (***e***). ***f***, ***g***, Po cell axonal boutons in MC or S1 at high light microscopic magnification. Note the size difference of axonal varicosities (white arrows). Scales bars: ***b***, 250 μm; ***c***, 50 μm; ***d***, ***e***, 100 μm; ***f***, ***g***, 5 μm.

For the 3D EM study, we labeled anterogradely with BDA relatively large populations of Po cell axons. Studies in rat indicate that MC and S1 may be targeted by axonal branches of the same Po neuron (Noseda et al., 2011; Ohno et al., 2012), making it unclear whether the structural differences we observed between the Po synapses in MC versus S1 reflect the existence either of two different Po cell populations, each projecting to one area, or of area-specific synapse structures in divergent axon branches of the same individual Po neurons. To address this question, isolated individual Po neurons were transfection-labeled with a Sindbis-pal-eGFP RNA construct using *in vivo* electroporation (Fig. 12). From a larger collection of fully-reconstructed Po neurons projecting to a variety of cortical territories (*n* = 12, data not shown), three cells were found to have an axonal branch specifically arborizing the vibrissal region of the MC; remarkably, all these three neurons had, in addition, a collateral axon branch arborizing in the vibrissal region of S1.

**Figure 13.**
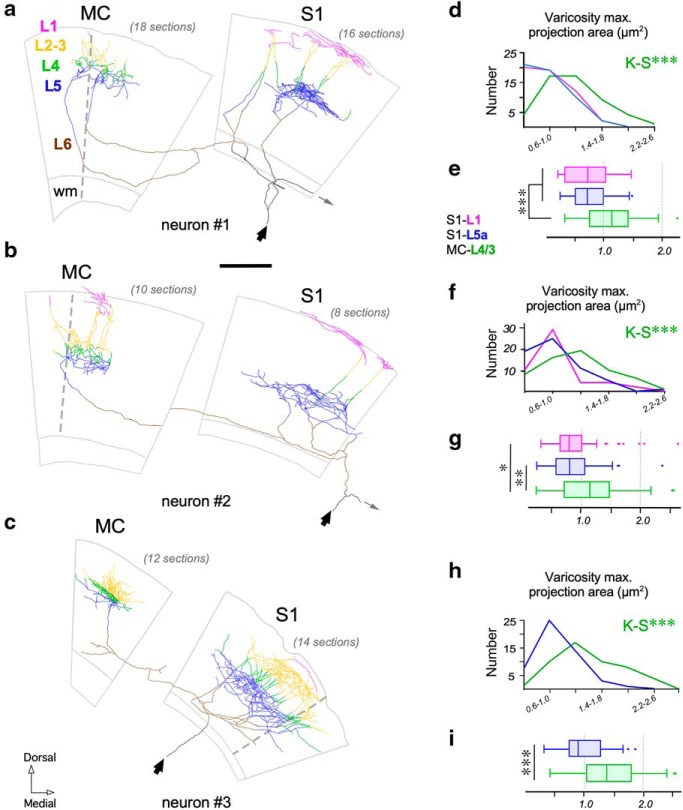
Individual branched Po axons target simultaneously MC and S1 and have varicosities of significantly different size in each area. ***a***–***c***, Camera Lucida reconstructions of the axonal arborizations of three individually-labeled Po neurons targeting both MC and S1. Axonal domains located in the different cortical layers or in the white matter (wm) are coded in different colors, as indicated. A dashed line in MC indicates the border between cytoarchitecnic areas Agl/M1 and Agm/M2 (Paxinos and Franklin, 2012). The point of entry of the axon arising from the thalamus is indicated by black arrows and the initial segment of a branch extending to more lateral areas (data not shown) by thin gray arrows, respectively. ***d***, ***f***, ***h***, Comparison of varicosity size (maximal projection areas) distributions of the MC L4/3 versus S1–L5a versus S1–L1 branches of each axon. Neuron 3 had virtually no axon branches in S1–L1. ***e***, ***g***, ***i***, Comparison of mean maximal projection areas. Mann–Whitney *U* Test. Scales bars, 500 μm. *Mostly small boutons, with occasional large boutons. **FIB-SEM data only.

The axonal arborizations in S1 and MC of three such cells are illustrated in Figure 13. The laminar distribution pattern of these individual axons in the cortex was equivalent to that produced by bulk-labeling with BDA iontophoresis (compare Figs. 1, 13). The Golgi-like quality of the Sindbis-Pal-eGFP glucose-oxidase immunostaining allowed a clear and sharp visualization of the somatodendritic domain and the full axonal arborization, including its varicosities. In each of the three Po neurons analyzed, the MC-L4/3 axonal varicosities were 1.5 to twofold larger (mean bouton projection areas: Neuron 1 = 1.13 ± 0.43 μm^2^, Neuron 2 = 1.13 ± 0.74 μm^2^, Neuron 3 = 1.42 ± 0.49 μm^2^) than those formed by the branches of the same axons in S1-L5a (0.74 ± 0.30, 0.83 ± 0.39, and 0.96 ± 0.35 μm^2^, respectively; *p* = 0.0026 for Neuron 1 and *p* < 0.001 for Neurons 2 and 3; Mann–Whitney test) or S1-L1 (0.73 ± 0.37 and 0.91 ± 0.46 μm^2^; *p* = 0.0117 for Neuron 1 and *p* < 0.001 for Neuron 2; Mann–Whitney test; Neuron 3 lacked axon branches in S1-L1; Fig. 13). Statistical comparison of size distributions revealed that the differences were also significant (S1L5a vs MC: *p* < 0.001 for all neurons; S1L5a vs MC: *p* = 0.002 for Neurons 1 and 2; Kolmogorov–Smirnov test; Fig. 13*d–i*). Hence, our single-cell analysis demonstrates that the Po cell axons form boutons in MC that are significantly larger than those formed by the same axon in S1.

## Discussion

**Figure 14.**
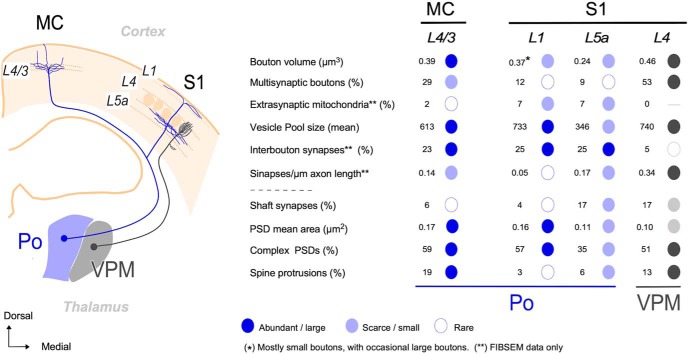
Graphic summary of relevant structural differences between Po axon synapses in MC-L4/3 and S1 (L1, L5a) and comparison with the S1–L4 VPM axon synapses. The diagram on the left depicts, on the outline of a coronal mouse brain section, the trajectory and cortical arborizations from a Po cell axon (blue) and a VPM cell axon (gray). The table on the right summarizes in simplified graphic format the most salient differences in synaptic structure between Po boutons in MC-L4/3, S1–L1, and S1–L5a (blue). The column on the right (gray) displays, with the same conventions, the values measured in S1–L4 VPM synapses. For clarity, numbers are rounded-up to mean values (for details and statistics, see Tables 1, 2; Fig. 9).

We have shown here that individual Po neurons simultaneously innervate MC and S1 through branched axons that in each area have varicosities (putative synaptic sites) of markedly different size. Fine-scale 3D-EM analysis revealed that the differences are even more pronounced at the ultrastructural level: the Po boutons in MC-L4/3 differ significantly from Po boutons in S1 (L1 and L5a) in volume, surface area and mean number of synaptic contacts. Moreover, the MC-L4/3 and S1-L5a Po synapses are markedly different in vesicle pool size, in PSD area and shape, and in the proportion of synapses established onto spines. A comparison with VPM S1-L4 synapses revealed both a sharp contrast between S1 Po and S1 VPM synapses, as well as intriguing similarities between MC Po synapses and S1 VPM synapses (Tables 1, 2; Fig. 14).

### Individual Po axons form structurally different synapses in MC and S1

Individual Po cell axons can innervate both the somatosensory and motor cortices, and often other regions (Noseda et al., 2011; Ohno et al., 2012). We recently showed that axonal varicosities in MC-L4/3 are, as a population, significantly larger than varicosities in S1-L5a (Casas-Torremocha et al., 2019). Here, using single-cell transfection-labeling of single Po neurons and quantitative axonal and bouton 3D-volume reconstructions, we demonstrate that the large MC-L4/3 and small S1–5a varicosities occur on separate branches of individual axons, and dovetail much deeper differences in synaptic 3D ultrastructure. To our knowledge, no previous study in mammals had examined the existence of differences in synapse structure and composition between distant branches of individual long-range projection axons.

Differences between Po MC-L4/3 axon synapses and S1-L5a in their mitochondrial volume, PSD area and number of synaptic vesicles are striking. Mitochondria docked at synaptic sites boost local ATP generation and control Ca^+2^ levels which allow enhanced mobilization and recycling of synaptic vesicles for exocytosis, neurotransmitter release and for the generation of synaptic membrane potentials, specially under repetitive high-frequency firing (Kharazia and Weinberg, 1994; Shepherd and Harris, 1998; Sheng and Cai, 2012; Cserép et al., 2018). Large vesicle pools and extensive and complex PSDs are associated with higher release probabilities and synaptic strength (Geinisman, 1993; Matz et al., 2010; Holderith et al., 2012; Valden et al., 2019) and to the number and distribution of postsynaptic at some synapses (Nusser et al., 1998; Ganeshina et al., 2004; Tarusawa et al., 2009). The Po MC-L4/3 boutons may thus more readily keep a high release probability at high-frequency firing rates than S1 boutons, consistent with the observed capacity of MC Po synapses to transmit signals with higher efficacy and temporal acuity than S1 Po synapses. In addition, specific glutamate receptor distributions may determine the divergent temporal profile of MC and S1 neuron responses to Po inputs (Casas-Torremocha et al., 2019; Mo and Sherman, 2019).

Thalamocortical shaft synapses in rodent neocortex correspond mostly to contacts on cortical inhibitory neurons (Staiger et al., 1996; but see Silver et al., 2003; Bopp et al., 2017). Whereas Po axons contact S1-L5a cortical interneurons in a proportion comparable to other thalamocortical systems and species (∼20%), we found far fewer dendritic shaft contacts in MC-L4/3 Po synapses, in register with the scarcity of VGluT2-positive boutons contacting dendritic shafts reported in mouse MC (Bopp et al., 2017). Thalamocortical axon activation of cortical interneurons produce a powerful disynaptic feedforward inhibition, in parallel with the excitation, on cortical neurons (Agmon and Connors, 1992; Gabernet et al., 2005; Cruikshank et al., 2007). A scarcity of Po shaft synapses seems thus consistent with the facilitation of MC and depression of S1 responses produced by rapid repetitive activation of Po axons (Casas-Torremocha et al., 2019).

### Po and VPM synapses in S1 are markedly different in structure

The Po and VPM synapses in adjacent layers of S1 involve postsynaptic shafts versus spines in identical proportion (17%), and have similar PSD sizes (Fig. 14). However, the presynaptic ultrastructure of the thalamic axons is strikingly different. Most (95%) VPM synaptic boutons are large, contain one or several mitochondria, large vesicle pools, and most of them (53%) have more than one (up to 4) active zones. Moreover, only 5% of the VPM synapses lack a mitochondrion (inter-bouton synapses), and virtually none contains free “nonsynaptic” mitochondria (White et al., 2004; Rodriguez-Moreno et al., 2018; Tables 1, 2; Fig. 14). Hence, in VPM axonal arbors, all mitochondria are docked near a synapse. In contrast, Po boutons in S1-L5a are ∼50% smaller, and have ∼30% smaller mitochondrial volumes and ∼50% smaller vesicle pools. Only 91% of them are monosynaptic, and none has more than two active zones. Importantly, ∼25% of Po PSDs occur in “non-varicose” axonal domains, without a mitochondrion nearby (< 2 μm), and ∼7% of the Po axon varicosities contain a mitochondrion but lack an active zone. Mitochondria in Po axons, therefore, are clearly far less concentrated around synaptic sites. This pattern resembles that of the hippocampal Shaffer collaterals, where 50% synapses lack a mitochondrion and 8% of mitochondria have no PSD nearby (Shepherd and Harris, 1998).

These differences may bear some relation with the recently discovered capacity of Po S1-L5a synapses for delayed yet stable potentiation as a result of conditional adult learning, a capacity that, surprisingly, is lacking in VPM S1-L4 synapses (Audette et al., 2019). During development, mitochondrial distribution along axonal trees is optimized to match the local metabolic demands of their synapses. However, a small but a significant fraction of mitochondria may remain mobile in adult axon arbors (for review, see Sheng and Cai, 2012). Low ATP and high calcium levels in highly active synapses promote the docking and/or fusion of mobile mitochondria (Saxton and Hollenbeck, 2012). In S1 Po axons, the strength of specific synapses might thus conceivably be modified in a relatively short time by the mobilization of some mitochondria. In contrast, the high basal mitochondria concentration at VPM S1 synapses may leave less room for synaptic strength modulation though mitochondrial redistribution.

In S1, Po and VPM synapses evoke each responses that have markedly different temporal profiles in cortical cells, in part because of the selective involvement of different glutamate receptors types: the VPM synapses involve only ionotropic receptors and their EPSCs depress markedly by repetitive stimulation (Lee and Sherman, 2008), whereas Po synapses involve both ionotropic and metabotropic receptors and display EPSCs-facilitation (Viaene et al., 2011; Casas-Torremocha et al., 2019). In addition to, the different presynaptic mitochondrial distribution in VPM versus Po synapses discussed above might contribute to their different effects on S1 neurons. By boosting local ATP production and Ca^2+^ buffering, axonal mitochondria promote the docking and/or fusion of synaptic vesicles (Sheng and Cai, 2012), as well as the mobilization of vesicles from the resting to the recycling and readily releasable pool (Esposito et al., 2012). As a result, mitochondria-rich synapses are capable of maintaining a high release probability over a wide range of firing rates and repetitive high-frequency firing (Cserép et al., 2018). A higher density of mitochondria at VPM S1 versus Po synapses seems thus consistent with electrophysiological evidence that VPM synapses produce relatively large (∼4.8 mV) EPSPs (Lee and Sherman, 2008) or currents (Petreanu et al., 2009) with low failure rates (Gil et al., 1999; Bruno and Sakmann, 2006), whereas Po synapses in S1-L5a produce smaller postsynaptic currents (Petreanu et al., 2009; Viaene et al., 2011; Mo and Sherman, 2019), which have slower rise and decay times (Bureau et al., 2006).

Despite arising from thalamic nuclei that are embryologically and functionally different (Clascá et al., 2016; Shi et al., 2017) and being located in separate cortical areas, the MC Po and S1-L4 VPM axon synapses show many structural similarities (Fig. 10; Tables 1, 2). This resemblance is consistent with observations that both elicit similarly large EPSCs, with paired-pulse depression (Lee and Sherman, 2008; Mo and Sherman, 2019) and involve only ionotropic receptors (Casas-Torremocha et al., 2019). Nevertheless, the Po MC-L4/3 synapse PSDs are 60% larger than VPM L4 PSDs, suggesting that they may have a higher synaptic strength when activated, in addition to being less tightly gated by feedforward inhibition, as discussed in the previous section (Casas-Torremocha et al., 2019).

In glutamatergic synapses, synaptic strength relates directly to ultrastructural parameters (Cserép et al., 2018). Although global postsynaptic cell or network effects depend on multiple variables and cannot be directly inferred form ultrastructure, the differences between Po synapses reported in the present study may be a significant contributor to the divergent responsivity and plasticity to Po input observed in MC versus S1 neurons (Casas-Torremocha et al., 2019; Mo and Sherman, 2019), as well as to the different responsivity and plasticity profiles of S1 neurons to VPM versus Po inputs (Petreanu et al., 2009; Audette et al., 2019).

On a broader perspective, the demonstration of branch-specific synaptic structure and dynamic properties in individual axons innervating separate cortical areas reveals an unsuspected subcellular level of complexity in thalamocortical circuits. This is a finding that upends current models of thalamocortical interaction (Jones, 1998; Sherman and Guillery, 2011) and that might as well illuminate the functional logic of other branched projection axon systems (Winnubst et al., 2019).
