# Supplementary material for: Area-Specific Synapse Structure in Branched Posterior Nucleus Axons Reveals a New Level of Complexity in Thalamocortical Networks
Source: J Neurosci. 2020 Mar 25;40(13):2663–79. doi: 10.1523/JNEUROSCI.2886-19.2020 (PMC7096142; doi:10.1523/JNEUROSCI.2886-19.2020)
Supplement: Table 1-1 [file zns999202369so1.docx]

**Table SM1. Tissue samples examined with electron microscopy and techniques applied**

|  | | ssTEM | | | | |  | | FIB-SEM | | | | |  |
| --- | --- | --- | --- | --- | --- | --- | --- | --- | --- | --- | --- | --- | --- | --- |
|  | |  | | | |  | |  | | | | |  |  |
|  | | S1L5a | | S1L1 | MCL3/4 | | |  | | S1L5a | S1L1 | MCL3/4 | | |
| *Mouse 1 R* | | 12 boutons | | 19 boutons | |  | |  | |  |  | |  | |
| *Mouse 2 R* | | 11 boutons | |  | |  | |  | | 10 boutons  108 linear µm |  | |  | |
| *Mouse 3 R* | | 25 boutons | | 44 boutons | | 25 boutons | |  | |  | 4 boutons  74 linear µm | |  | |
| *Mouse 4 L* | | 16 boutons | |  | | 17 boutons | |  | |  |  | | 9 boutons  101 linear µm | |
